# Extrasynaptic Communication

**DOI:** 10.3389/fnmol.2021.638858

**Published:** 2021-04-30

**Authors:** Francisco F. De-Miguel, Carolina Leon-Pinzon, Susana G. Torres-Platas, Vanessa del-Pozo, Guillermo A. Hernández-Mendoza, Dilia Aguirre-Olivas, Bruno Méndez, Sharlen Moore, Celeste Sánchez-Sugía, Marco Antonio García-Aguilera, Alejandro Martínez-Valencia, Guillermo Ramírez-Santiago, J. Miguel Rubí

**Affiliations:** ^1^Instituto de Fisiología Celular-Neurociencias, Universidad Nacional Autónoma de México, México City, Mexico; ^2^Centro de Ciencias de la Complejidad, Universidad Nacional Autónoma de México, México City, Mexico; ^3^Posgrado en Ciencias Físicas, Universidad Nacional Autónoma de México, México City, Mexico; ^4^Instituto de Matemáticas, Universidad Nacional Autónoma de México, Juriquilla, Mexico; ^5^Facultat de Fisica, Universitat de Barcelona, Barcelona, Spain

**Keywords:** serotonin, extrasynaptic release, modulation, nerve cell communication, somatic exocytosis, glia

## Abstract

Streams of action potentials or long depolarizations evoke a massive exocytosis of transmitters and peptides from the surface of dendrites, axons and cell bodies of different neuron types. Such mode of exocytosis is known as extrasynaptic for occurring without utilization of synaptic structures. Most transmitters and all peptides can be released extrasynaptically. Neurons may discharge their contents with relative independence from the axon, soma and dendrites. Extrasynaptic exocytosis takes fractions of a second in varicosities or minutes in the soma or dendrites, but its effects last from seconds to hours. Unlike synaptic exocytosis, which is well localized, extrasynaptic exocytosis is diffuse and affects neuronal circuits, glia and blood vessels. Molecules that are liberated may reach extrasynaptic receptors microns away. The coupling between excitation and exocytosis follows a multistep mechanism, different from that at synapses, but similar to that for the release of hormones. The steps from excitation to exocytosis have been studied step by step for the vital transmitter serotonin in leech Retzius neurons. The events leading to serotonin exocytosis occur similarly for the release of other transmitters and peptides in central and peripheral neurons. Extrasynaptic exocytosis occurs commonly onto glial cells, which react by releasing the same or other transmitters. In the last section, we discuss how illumination of the retina evokes extrasynaptic release of dopamine and ATP. Dopamine contributes to light-adaptation; ATP activates glia, which mediates an increase in blood flow and oxygenation. A proper understanding of the workings of the nervous system requires the understanding of extrasynaptic communication.

## Introduction

Our view of the workings of the nervous system have been dominated by four threads of fundamental evidence: First, Cajal defined nerve circuits as networks of neurons connected in stereotyped manner, forming transmission lines for specific information processing. Second, physiologists such as Helmholtz, Hodgkin and Huxley showed that nerve impulses spread along axons at ~300 km/h. Third, Sherrington, Katz, Kuffler and Eccles demonstrated that synapses transmit information in ~0.5 ms. Fourth, plasticity adapts synaptic transmission to variations in the ongoing pattern of electrical activity. Such conceptual framework explains how a table tennis player detects the trajectory and velocity of a ball approaching at 50 Km/h and in ~0.3 ms and coordinates his whole-body motion to send it back to an opposite corner of the table. In the games, such cycles may occur twice per second!

This review article deals with a parallel form of communication: streams of electrical impulses or long depolarizations promote massive liberation of signaling molecules from certain neurons. Release occurs without use of synaptic structures, therefore, it is named extrasynaptic. Molecules that are released extrasynaptically from the soma, dendrites and axon modulate the responses of entire neuronal circuits from seconds to days (Trueta and De-Miguel, 2012). Such form of communication may explain why a table tennis player is defeated after being left by his fiancée. His reduced concentration, motivation and attention make him react poorly during the game. A hypothesis gaining increasing support is that ranges of physiological concentrations of extracellular signaling molecules modulate the responses of whole neuronal circuits; concentrations below or above produce pathologies (Calabresi et al., 2015; Del-Bel and De-Miguel, 2018; Pál, 2018; Quentin et al., 2018).

Our focus here is exclusively on molecules that are released by exocytosis. Other substances such as nitric oxide or cannabinoids are released by diffusion across the plasma membrane (Del-Bel and De-Miguel, 2018); nucleic acids and proteins are released encapsulated inside vesicles that flow extracellularly (Colombo et al., 2014; Mendolesi, 2018). Those forms of release also follow increases in electrical activity.

A good example as to how extrasynaptic exocytosis exerts its effects comes from studies of aggression in lobsters by Kravitz and his colleagues (Kravitz, 1988; Huber et al., 1997). An encounter between two lobsters triggers aggression. Lobsters approximate to each other displaying their powerful claws and urinating on each other. The episodic encounters, initially lasting seconds, decrease their strength and duration as one lobster becomes dominant and the other submissive. The aggressive posture is evoked by a systemic injection of serotonin; an injection of octopamine reproduces the submissive posture (Livingstone et al., 1980).

The serotonergic A1 neurons in the abdominal ganglia of lobsters innervate the ganglia and project branches to the circulation. Command neurons that evoke tail flipping during aggression evoke serotonin release from the A1 neurons. Serotonin that is released in the ganglia lowers the firing threshold of central neurons; the serotonin discharged to the circulation increases motoneuron transmission and strengthens muscle contractions and heart beat (Glusman and Kravitz, 1982; Hörner et al., 1997; Hernández-Falcón et al., 2005). In experiments in which serotonergic neurons were depleted of serotonin by systemic injection of 5–7 dihydroxytryptamine, aggression still occurred. However, the strength and duration of the encounters lacked modulation. Therefore, serotonin “sets the gain” of the circuitry for aggression by acting all along the neuronal circuit.

### Cellular Basis of Extrasynaptic Exocytosis

Extrasynaptic exocytosis has been studied in central and peripheral neurons. The similitude among the mechanism that links excitation with exocytosis suggests a widely conserved mechanism, similar to that in gland cells but remarkably different from that for synaptic release (Sun and Poo, 1987; Huang et al., 2012; Hirasawa et al., 2015; Ludwig and Stern, 2015; Hökfelt et al., 2018; Quentin et al., 2018). Most transmitters and all peptides have been shown to be released extrasynaptically (Trueta and De-Miguel, 2012), and neurons may release more than one type of substance (Burnstock, 2012; Nusbaum et al., 2017; Hökfelt et al., 2018).

### Extrasynaptic Exocytosis From Different Neuronal Compartments

An evolutionary feature shared by neurons that release extrasynaptically is that small numbers innervate the nervous system extensively, and produce a wide variety of effects. For example, ~235,000 serotonergic neurons in humans (Baker et al., 1990) project from the raphe nuclei to the entire central nervous system. Neurons releasing catecholamines, acetylcholine or peptides exist in similar small numbers (Zetler, 1970; Mouton et al., 1994; Nair-Roberts et al., 2008; Li et al., 2018). Such extensive innervation is complemented by the neuronal capability to release differentially from the soma, dendrites and axon. A well-known example is the release of the peptides vasopressin or oxytocin from magnocellular hypothalamic neurons (Ludwig and Stern, 2015).

The axons of magnocellular neurons bear rosaries of varicosities that release extrasynaptically on the spread of action potentials; their terminals discharge peptide onto the blood flow (Acher and Chauvet, 1954; Du Vigneaud, 1954). During lactation, suckling evokes oxytocin axonal release but dendritic release is delayed. However, dendritic release is locally evoked on activation of extrasynaptic NMDA receptors (de Kock et al., 2004; Tobin et al., 2012), as it also happens in dendrites of raphe neurons (Colgan et al., 2012).

### Discovery of Extrasynaptic Communication

Serotonin that had been released extrasynaptically was discovered by Dalstrom and Fuxe in the 60s using the Falck-Hillarp technique, by which exposure to formaldehyde vapors transforms the monoamines serotonin, dopamine or adrenaline into fluorescent derivatives (Fuxe et al., 2007; Borroto-Escuela et al., 2015). Brain sections of raphe nuclei contained serotonin-derived fluorescence surrounding the fluorescent cell bodies, distantly from the axonal release sites. Similar observations made in dopaminergic neurons, plus the fact that peptides can be released far away from their receptors led to the concept of volume transmission by Fuxe and his colleagues, meaning that molecules act on receptors located distantly from the release sites (Borroto-Escuela et al., 2015). It was later shown that axons of neurons releasing monoamines, acetylcholine, ATP and peptides bear vesicle arrangements but scarce presynaptic active zones. Therefore, most exocytosis occurs extrasynaptically (Hökfelt, 1968; Umbriaco et al., 1995; Contant et al., 1996; Descarries et al., 1996; Descarries and Mechawar, 2000; Burnstock, 2012).

### Somatic Release of Serotonin

The vast diversity and distribution of serotonin functions, the small numbers of serotonergic neurons and synapses, and the extraordinary chemical properties of serotonin explain why extrasynaptic serotonergic communication has been widely studied. Serotonin that is released from the cell body and dendrites of raphe neurons has been detected distantly by voltammetry, based on its redox properties (Bunin and Wightman, 1998). Moreover, serotonin exocytosis has been detected by amperometric electrodes apposed onto the soma of leech Retzius neurons (Bruns et al., 2000), or in the soma and dendrites of raphe neurons by multiphoton excitation (Kaushalya et al., 2008; Colgan et al., 2012; Sarkar et al., 2012; Maity and Maiti, 2018).

The mechanism for somatic exocytosis of serotonin has been studied step by step in Retzius neurons (De-Miguel et al., 2012). Their large (60–80 μm) soma contains “astronomic” numbers of dense-core vesicles loaded with serotonin (Coggeshall, 1972). Most vesicles rest distantly from the plasma membrane. However, electron microscopy and fluorescence of FM dyes, which stain the intravesicular membrane during exo-endocytosis (Hoopmann et al., 2012), indicate that vesicles move massively to the plasma membrane following trains of action potentials but not individual impulses (Trueta et al., 2003). The formation of fluorescent spots beneath the soma surface indicates that fusion of dense-core vesicles occurs in preferential sites. The development of FM fluorescent spots reflects the kinetics of release by clusters of vesicles; the number of fluorescent spots is a measure of the amount of release. Such experiments gave unexpected results: First, exocytosis starts seconds after the end of stimulation. Second, exocytosis lasts hundreds of seconds (Trueta et al., 2003), as hormone release from gland cells does (Thorn et al., 2016). Similar results have been obtained from cholinergic, dopaminergic, noradrenergic and peptidergic neurons (Sun and Poo, 1987; Huang and Neher, 1996; Jaffe et al., 1998; Puopolo et al., 2001; Bao et al., 2003; Kaushalya et al., 2008; Huang et al., 2012; Ludwig and Stern, 2015).

In Retzius neurons and magnocellular neurons it is the frequency of the action potentials, not their number, what determines the amount of release. The maximum release occurs at 20 impulses per second (Dreifuss et al., 1971; Leon-Pinzon et al., 2014), but may be enhanced by alternate periods of stimulation and rest (Dutton and Dyball, 1979).

### Vesicle Transport

The delay between stimulation and exocytosis reflects the vesicle transport to the plasma membrane (De-Miguel et al., 2012). The large calcium transient that develops upon the stimulation train is essential for the vesicle transport. In electron micrographs, vesicles remain at rest upon stimulation with extracellular magnesium substituting for calcium to block calcium entry. Moreover, experimental perturbations of the tubulin-kinesin or actin-myosin transport systems prevent somatic exocytosis from Retzius and magnocellular neurons (Tobin and Ludwig, 2007; De-Miguel et al., 2012; Noguez et al., 2019).

**Figure 1:**
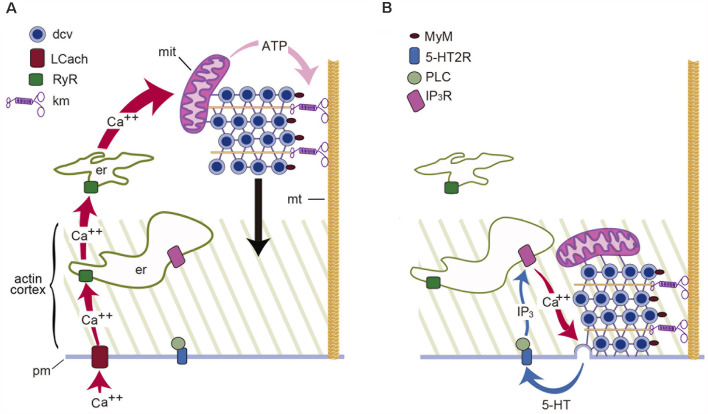
Schematic representation of the mechanism for somatic exocytosis of serotonin in Retzius neurons. **(A)** Electrical activity sets in motion the transport of dense core vesicles (dcv) to the plasma membrane. In response to a train of action potentials, L-type calcium channels (LCach) open. Calcium entry activates ryanodine receptors (RyR) in the endoplasmic reticulum (er) and produces calcium-induced calcium release. The amplified calcium wave invades the soma; in the mitochondria (mit), calcium stimulates the synthesis of ATP, which activates kinesin motors (km) and vesicle transport along microtubules (mt). **(B)** Vesicles enter the actin cortex and myosin motors (MyM) carried by the vesicles couple to actin filaments and contribute to the transport. Release is maintained by a positive feedback loop in which the serotonin that is released activates 5-HT2 receptors (5-HT2R). Activation of phospholipase C (PLC) produces IP_3_ which acts on its receptors (IP_3_R) to activate calcium release. Such calcium maintains exocytosis going on until the last vesicles fuse (Adapted from Leon-Pinzon et al., 2014).

Stimulation and the activation of the transport are linked by a chain of events: first, stimulation promotes calcium entry through L-type channels (Trueta et al., 2003), which are advantageous, for their slow inactivation sustains calcium entry along trains of impulses or long depolarizations. Second, increasing the stimulation frequency increases the amplitude of the intracellular calcium transient (Leon-Pinzon et al., 2014). Third, calcium activates ryanodine receptors, inducing calcium-dependent calcium release (Trueta et al., 2004; Leon-Pinzon et al., 2014), as in substantia nigra of dopaminergic and magnocellular neurons (Ludwig et al., 2002; Patel et al., 2009). Fourth, a calcium tsunami floods the cell body and invades the mitochondria, which respond by producing ATP. Fifth, ATP sets in motion the kinesin- and myosin-dependent vesicle transport (Figure 1; De-Miguel et al., 2012).

### Energy Cost of the Vesicle Transport

Application of thermodynamic theory to the kinetics of exocytosis predicts that three variables determine the latency from stimulation to the onset of exocytosis (De-Miguel et al., 2012 ): the traveling distance to the plasma membrane (0.2–6.0 μm), the velocity of the transport (15–90 nm/s) and the number of vesicles carried per cluster (90 to >1,000). Upon arrival at the plasma membrane, vesicles fuse at a 0.5–4.0 s^−1^ rate, which reflects the transport velocity. For example, exocytosis from 1,000 vesicles at a 4 s^−1^ rate lasts 250 s. The energy expenses of the transport, calculated from the work of the motors, range from 10–200 ATP molecules per vesicle fused, depending on the same variables (De-Miguel et al., 2012).

How thermodynamically-efficient is the use of ATP during the vesicle transport? An answer has been obtained also from the application of thermodynamic theory (Noguez et al., 2019). Surprisingly, the largest thermodynamic efficiency value is 6.2%, which is lower than the 20% efficiency of a contemporary car running on a highway. The remaining energy is dissipated as heat along the path, owing to friction forces. The origin of such friction was predicted from the distribution of efficiency values along the traveling pathway. The lowest values correlate with the penetration of vesicles to the actin cortex and their passage between endoplasmic reticulum layers. Both essential contributors to the transport increase the energy cost by being frictive obstacles. Such a phenomenon adds energy cost to the modulation of neuronal circuits.

### Calcium and Exocytosis

Measurements of the intracellular calcium dynamics with fluorescent dyes unveiled that by the time vesicles arrive at the plasma membrane, the intracellular calcium concentration has returned to resting levels except in the soma shell (Leon-Pinzon et al., 2014). Such peripheral calcium elevation drives the fusion of vesicles as they arrive at the plasma membrane. Voltage clamp measurements failed to detect any transmembrane calcium flow following the train of impulses. Instead, the peripheral calcium transient was reproduced by iontophoretic serotonin application to activate membrane receptors. Conclusive evidence that the peripheral calcium transient depends on serotonin that had been released came from experiments in which both the peripheral calcium transient and somatic exocytosis were prevented by blocking serotonergic 5HT2 receptors with methysergide, or by blocking the activation of phospholipase C (PLC) with U-73122 before stimulation of somatic exocytosis (Leon-Pinzon et al., 2014). Therefore, the calcium that sustains exocytosis is released by peripheral endoplasmic reticulum upon a serotonin-mediated IP_3_ production and activation of IP_3_ receptors. Similar observations made in peptidergic dorsal root ganglion and magnocellular neurons (Bao et al., 2003; Ludwig and Stern, 2015) point to another general principle: the long-lasting exocytosis is sustained by a feedback loop. Transmitter that is liberated activates autoreceptors coupled to phospholipase C; IP_3_ production evokes intracellular calcium release; calcium promotes exocytosis; the released substance maintains the cycle. Termination of the loop follows the fusion of the last vesicles in the clusters (Figure 1).

### Molecules Catalyzing Exocytosis

Synapses contain a calcium-sensitive molecular complex that drives vesicle fusion upon local calcium elevations (Südhof, 2013). In the soma and dendrites of dopaminergic neurons, antibodies recognize isoforms of the fusion complex components VAMP-2, SNAP25, and syntaxin, which are unusual at synapses (Witkovsky et al., 2009). Moreover, the synaptic calcium sensors synaptotagmins 1 and 2 are substituted by the more appropriate high affinity synaptotagmins 4 and 7 (Mendez et al., 2011), since the fusion of dense-core vesicles depends on distant calcium sources.

### Dense-Core Vesicle Recycling

The vesicle recycling has been studied by adding the marker peroxidase to the extracellular fluid before stimulation (Trueta et al., 2012). Sections for electron microscopy incubated with anti-peroxidase antibody coupled to gold particles, showed peroxidase inside dense-core vesicles, newly-formed small (~40 nm) clear vesicles, and inside multivesicular bodies containing both vesicle types (Figure 1). Multivesicular bodies are transported retrogradely and their content is recycled in the perikaryon to form new vesicles.

### Synaptic vs. Extrasynaptic Exocytosis

Studies in Retzius neurons permit a comparison of the amounts of transmitter liberated from synaptic and extrasynaptic vesicle pools. The formation of specific synapses between identified leech neurons in culture allowed John Nicholls and his colleagues (Nicholls and Kuffler, 1990) to examine the fine mechanisms of transmission. At synapses, the fusion of clear vesicles is calcium-dependent. Amperometric records show that a vesicle liberates ~4,700 serotonin molecules (Bruns and Jahn, 1995). Synapses display short-term plasticity (Stewart et al., 1989); for example, 10 impulses at 20-Hz evoke rapid facilitation followed by depression, along which ~60 quanta get released.

**Figure 2:**
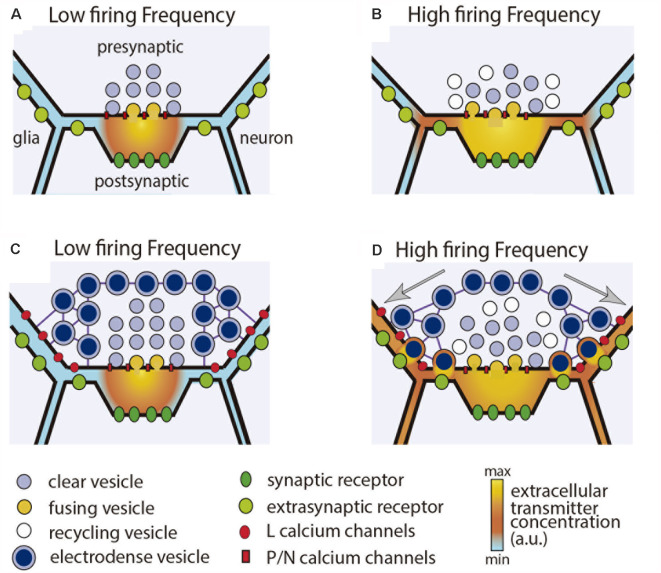
Synaptic and perisynaptic exocytosis. **(A)** Low frequency stimulation activates presynaptic P or N type calcium channels and evokes fusion of synaptic vesicles in the active zone. Transmitter liberated into the synaptic cleft activates post-synaptic receptors. **(B)** Increasing the stimulation frequency increases synaptic exocytosis and produces transmitter spillover, which acts on extrasynaptic receptors in adjacent glia and neuronal processes. **(C)** In the presence of perisynaptic dense-core vesicles, a low stimulation frequency evokes mostly synaptic release. **(D)** Increasing the stimulation frequency produces summation of calcium currents flowing through L type channels. Dense core vesicles are transported to the plasma membrane and fuse in presynaptic regions of the terminal. Transmitter that has been released acts on extrasynaptic receptors in the pre-and post-synaptic terminals, and also in adjacent glial and neuronal processes. Clear and dense core vesicles may have the same or different transmitters. Dense core vesicles also may contain peptides.

A common form of extrasynaptic exocytosis of transmitters and peptides occurs from dense-core vesicles surrounding synaptic active zones (Hökfelt et al., 2018). The differences between synaptic and perisynaptic exocytosis are schematized in Figure 2. The presynaptic boutons of cultured Retzius neurons contain clear synaptic vesicles and dense-core perisynaptic vesicles all filled with serotonin (Kuffler et al., 1987; Bruns and Jahn, 1995). Perisynaptic release increases along stimulation trains and produces large quantal amperometric spikes upon release of ~90,000 serotonin molecules (Bruns and Jahn, 1995). Amazingly, three dense-core vesicles release about the same number of serotonin molecules as the 60 synaptic vesicles that fuse along a 20-Hz train.

The difference between synaptic and somatic exocytosis is more drastic. Electron microscopy and FM dye staining of vesicles indicate that a 20-Hz train evokes fusion of ~40,000 vesicles from ~80 release sites, each vesicle cluster carrying on an average 500 vesicles (Del-Bel and De-Miguel, 2018). By assuming that ~90,000 serotonin molecules integrate a quantum, a 10-impulse train at 20-Hz would trigger release of ~3.6 billion molecules. Moreover, the long thick axon discharges serotonin from clear and dense-core vesicles in undetermined amounts. It is predictable that the huge amount of serotonin being released from a pair of Retzius cells in each ganglion suffices to modulate behavior (Willard, 1981).

### Transmitter Spillover

Glutamate and GABA, the conventional transmitters at synapses, act extrasynaptically upon spillover from the synaptic cleft when synaptic release increases (Isaacson et al., 1993; Rusakov and Kullmann, 1998; DiGregorio et al., 2002). Spillover-mediated transmission occurs through activation of low-affinity extrasynaptic receptors in neighboring cells (Pál, 2018). In addition, astroglia and microglia sense and release glutamate (Pál, 2018).

### Glia as Mediator of Extrasynaptic Communication

Glia are common counterparts for extrasynaptic exocytosis. Glial cell membranes respond to transmitters and transport many of them (Marcaggi and Attwell, 2004; Káradóttir et al., 2005; Verkhratsky et al., 2009). In response to transmitters such as glutamate, serotonin and ATP, networks of electrically-coupled astrocytes propagate calcium transients (Munsch and Deitmer, 1992; Metea and Newman, 2006; Verkhratsky et al., 2009). In return, glia releases the same or other transmitters, peptides and proteins (Billups and Attwell, 1996; Henneberger et al., 2010; Igelhorst et al., 2015). Observations like these have led to the hypothesis of tripartite synapses, in which glia reacts to transmitters that spillover and in return modulate synaptic activity (Perea et al., 2009; Corkrum et al., 2020).

### Extrasynaptic Integration of Retinal Responses to Light

The retina provides a clear example of the integrative roles of extrasynaptic communication at the cellular level. A light spot shone onto a dark-adapted retina, evokes visual processing and extrasynaptic release of transmitters from amacrine cells (Hirasawa et al., 2015; Newman, 2015). Dopamine contributes to light adaptation by uncoupling electrical synapses and by acting directly on neurons at every level of the visual processing (Piccolino et al., 1984; Witkovsky, 2004; Zhang et al., 2011). Solid evidence about glia as mediator between extrasynaptic exocytosis and the regulation of blood flow has been contributed by Newman and his colleagues (Newman, 2015). ATP released from Amacrine cells activates Muller cells, the main type of retinal glia. In response, Muller cells synthesize and release factors that increase blood flow and oxygenation of the illuminated area. By extrapolation, the magnetic resonance images may be a product of extrasynaptic communication.

## Conclusions

1. Extrasynaptic exocytosis is common in the nervous system. It may occur differentially from the soma, dendrites and axon, allowing neurons to produce multiple effects.
2. Synaptic and extrasynaptic exocytosis coexist in the same neurons.
3. Neurotransmitters and peptides are released extrasynaptically.
4. Synaptic transmission is punctual; extrasynaptic transmission is diffuse. Substances released extrasynaptically act *via* volume transmission at variable distances and with different time courses.
5. Extrasynaptic communication integrates the activity of neurons, glia and blood vessels.
6. Other forms of extrasynaptic neurotransmission occur upon diffusive release of molecules, such as gases and cannabinoids; vesicles are released loaded with cocktails of molecules.
7. Understanding the functioning of the nervous system requires understanding of its modulation by extrasynaptic communication.

## Author Contributions

FD-M wrote the first version of the manuscript. All authors contributed to the article and approved the submitted version.

## Conflict of Interest

The authors declare that the research was conducted in the absence of any commercial or financial relationships that could be construed as a potential conflict of interest.

## References

[B1] Acher R. Chauvet J. (1954). The structure of bovine vasopressin. Biochim. Biophys. Acta. 14, 421–429. 10.1016/0006-3002(54)90202-4 13181900

[B2] Baker K. G. Halliday G. M. Törk I. (1990). Cytoarchitecture of the human dorsal raphe nucleus. J. Comp. Neurol. 8, 147–161. 10.1002/cne.903010202 2262589

[B3] Bao L. Jin S. X. Zhang C. Wang L. H. Xu Z. Z. Zhang F. X.. (2003). Activation of delta opioid receptors induces receptor insertion and neuropeptide secretion. Neuron 37, 121–133. 10.1016/s0896-6273(02)01103-0 12526778

[B5] Billups B. Attwell D. (1996). Modulation of non-vesicular glutamate release by pH. Nature 379, 171–174. 10.1038/379171a0 8538768

[B6] Borroto-Escuela D. O. Agnati L. F. Bechter K. Jansson A. Tarakanov A. O. Fuxe K. (2015). The role of transmitter diffusion and flow versus extracellular vesicles in volume transmission in the brain neural-glial networks. Philos. Trans. R. Soc. Lond. B Biol. Sci. 370:20140183. 10.1098/rstb.2014.0183 26009762 PMC4455752

[B8] Bruns D. Jahn R. (1995). Real-time measurement of transmitter release from single synaptic vesicles. Nature 377, 62–65. 10.1038/377062a0 7659162

[B7] Bruns D. Riedel D. Klingauf J. Jahn R. (2000). Quantal release of serotonin. Neuron 28, 205–220. 10.1016/s0896-6273(00)00097-0 11086995

[B9] Bunin M. A. Wightman R. M. (1998). Quantitative evaluation of 5-hydroxytryptamine (serotonin). neuronal release and uptake: an investigation of extrasynaptic transmission. J. Neurosci. 18, 4854–4860. 10.1523/JNEUROSCI.18-13-04854.1998 9634551 PMC6792557

[B10] Burnstock G. (2012). Purinergic signalling: its unpopular beginning, its acceptance and its exciting future. Bioessays 34, 218–225. 10.1002/bies.201100130 22237698

[B11] Calabresi P. Ghiglieri V. Mazzocchetti P. Corbelli I. Picconi B. (2015). Levodopa-induced plasticity: a double-edged sword in Parkinson’s disease. Philos. Trans R Soc. Lond B Biol. Sci. 370:20140184. 10.1098/rstb.2014.0184 26009763 PMC4455753

[B12] Coggeshall R. E. (1972). Autoradiographic and chemical localization of 5-hydroxytryptamine in identified neurons in the leech. Anat. Rec. 172, 489–498. 10.1002/ar.1091720303 4536825

[B13] Colgan L. A. Cavolo S. L. Commons K. G. Levitan E. S. (2012). Action potential-independent and pharmacologically unique vesicular serotonin release from dendrites. J. Neurosci. 32, 15737–15746. 10.1523/JNEUROSCI.0020-12.2012 23136413 PMC3505755

[B14] Colombo M. Raposo G. Théry C. (2014). Biogenesis, secretion and intercellular interactions of exosomes and other extracellular vesicles. Annu. Rev. Cell Dev. Biol. 30, 255–289. 10.1146/annurev-cellbio-101512-122326 25288114

[B15] Contant C. Umbriaco D. Garcia S. Watkins K. C. Descarries L. (1996). Ultrastructural characterization of the acetylcholine innervation in adult rat neostriatum. Neuroscience 71, 937–947. 10.1016/0306-4522(95)00507-2 8684624

[B16] Corkrum M. Covelo A. Lines J. Bellocchio L. Pisansky M. Loke K.. (2020). Dopamine-evoked synaptic regulation in the nucleus accumbens requires astrocyte activity. Neuron 105, e5.1036–e5.1047. 10.1016/j.neuron.2019.12.026 31954621 PMC7322729

[B100] de Kock C. P. J. Burnashev N. Lodder J. C. Mansvelder H. D. Brussaard A. B. (2004). NMDA receptors induce somatodendritic secretion in hypothalamic neurones of lactating female rats. J. Physiol. 561, 53–64. 10.1113/jphysiol.2004.069005 15459239 PMC1665332

[B17] Del-Bel E. De-Miguel F. F. (2018). Extrasynaptic neurotransmission mediated by exocytosis and diffusive release of transmitter substances. Front. Synaptic Neurosci. 10:13. 10.3389/fnsyn.2018.00013 29937726 PMC6003215

[B18] De-Miguel F. F. Santamaría-Holek I. Noguez P. Bustos C. Hernández-Lemus E. Rubí J. M. (2012). Biophysics of active vesicle transport, an intermediate step that couples excitation and exocytosis of serotonin in the neuronal soma. PLoS One 7:e45454. 10.1371/journal.pone.0045454 23056204 PMC3463611

[B20] Descarries L. Mechawar N. (2000). Ultrastructural evidence for diffuse transmission by monoamine and acetylcholine neurons of the central nervous system. Prog. Brain Res. 125, 27–47. 10.1016/S0079-6123(00)25005-X 11098652

[B19] Descarries L. Watkins K. C. Garcia S. Bosler O. Doucet G. (1996). Dual character, asynaptic and synaptic, of the dopamine innervation in adult rat neostriatum: a quantitative autoradiographic and immunocytochemical analysis. J. Comp. Neurol. 375, 167–186. 10.1002/(SICI)1096-9861(19961111)375:2<167::AID-CNE1>3.0.CO;2-0 8915824

[B21] DiGregorio D. A. Nusser Z. Silver R. A. (2002). Spillover of glutamate onto synaptic AMPA receptors enhances fast transmission at a cerebellar synapse. Neuron 35, 521–533. 10.1016/s0896-6273(02)00787­0 12165473

[B101] Dreifuss J. J. Kalnins I. Kelly J. S. Ruf K. B. (1971). Action potentials and release of neurohypophysial hormones *in vitro*. J. Physiol. 215, 805–817. 10.1113/jphysiol.1971.sp009499 4326309 PMC1331915

[B22] Du Vigneaud V. (1954). Hormones of the posterior pituitary gland: oxytocin and vasopressin. Harvey Lect. 50, 1–26. 13306033

[B23] Dutton A. Dyball R. E. (1979). Phasic firing enhances vasopressin release from the rat neurohypophysis. J. Physiol. 290, 433–440. 10.1113/jphysiol.1979.sp012781 469785 PMC1278845

[B24] Fuxe K. Dahlström A. Höistad M. Marcellino D. Jansson A. Rivera A.. (2007). From the Golgi-Cajal mapping to the transmitter-based characterization of the neuronal networks leading to two modes of brain communication: wiring and volume transmission. Brain Res. Rev. 55, 17–54. 10.1016/j.brainresrev.2007.02.009 17433836

[B25] Glusman S. Kravitz E. A. (1982). The action of serotonin on excitatory nerve terminals in lobster nerve-muscle preparations. J. Physiol. 325, 223–241. 10.1113/jphysiol.1982.sp014147 6125589 PMC1251391

[B28] Hökfelt T. (1968). *In vitro* studies on central and peripheral monoamine neurons at the ultrastructural level. Z Zellforsch Mikrosk Anat. 91, 1–74. 10.1007/BF00336984 5724812

[B27] Hökfelt T. Barde S. Xu Z. D. Kuteeva E. Rüegg J. Le Maitre E.. (2018). Neuropeptide and small transmitter coexistence: fundamental studies and relevance to mental illness. Front. Neural Circuits 12:106. 10.3389/fncir.2018.00106 30627087 PMC6309708

[B29] Hörner M. Weiger W. A. Edwards D. H. Kravitz E. A. (1997). Excitation of identified serotonergic neurons by escape command neurons in lobsters. J. Exp. Biol. 200, 2017–2033. 9246785 10.1242/jeb.200.14.2017

[B30] Henneberger C. Papouin T. Oliet S. H. Rusakov D. A. (2010). Long-term potentiation depends on release of D-serine from astrocytes. Nature 463, 232–236. 10.1038/nature08673 20075918 PMC2807667

[B31] Hernández-Falcón J. Basu A. C. Govindasamy S. Kravitz E. A. (2005). Changes in heart rate associated with contest outcome in agonistic encounters in lobsters. Cell Mol. Neurobiol. 25, 329–343. 10.1007/s10571-005-3063-x 16047545 PMC11529612

[B32] Hirasawa H. Contini M. Raviola E. (2015). Extrasynaptic release of GABA and dopamine by retinal dopaminergic neurons. Philos Trans. R Soc. Lond B Biol. Sci. 370:20140186. 10.1098/rstb.2014.0186 26009765 PMC4455755

[B33] Hoopmann P. Rizzoli S. O. Betz W. J. I. (2012). Imaging synaptic vesicle recycling by staining and destaining vesicles with FM dyes. Cold Spring Harb. Protoc. 2012, 77–83. 10.1101/pdb.prot067603 22194270

[B34] Huang L. Y. Neher E. (1996). Ca^2+^-dependent exocytosis in the somata of dorsal root ganglion neurons. Neuron 17, 135–145. 10.1016/s0896-6273(00)80287-1 8755485

[B35] Huang H. P. Zhu F. P. Chen X. W. Xu Z. Q. Zhang C. X. Zhou Z. (2012). Physiology of quantal norepinephrine release from somatodendritic sites of neurons in locus coeruleus. Front. Mol. Neurosci 5:29. 10.3389/fnmol.2012.00029 22408604 PMC3295224

[B36] Huber R. Orzeszyna M. Pokorny N. Kravitz E. A. (1997). Biogenic amines and aggression: experimental approaches in crustaceans. Brain Behav. Evol. 50, 60–68. 10.1159/000113355 9217993

[B37] Igelhorst B. A. Niederkinkhaus V. Karus C. Lange M. D. Dietzel I. D. (2015). Regulation of neuronal excitability by release of proteins from glial cells. Philos Trans. R Soc. Lond B Biol. Sci. 370:20140194. 10.1098/rstb.2014.0194 26009773 PMC4455763

[B38] Isaacson J. S. Solís J. M. Nicoll R. A. (1993). Local and diffuse synaptic actions of GABA in the hippocampus. Neuron 10, 165–175. 10.1016/0896-6273(93)90308-e 7679913

[B39] Jaffe E. H. Marty A. Schulte A. Chow R. H. (1998). Extrasynaptic vesicular transmitter release from the somata of substantia nigra neurons in rat midbrain slices. J. Neurosci. 18, 3548–3553. 10.1523/JNEUROSCI.18-10-03548.1998 9570786 PMC6793140

[B40] Káradóttir R. Cavelier P. Bergersen L. H. Attwell D. (2005). NMDA receptors are expressed in oligodendrocytes and activated in ischaemia. Nature 438, 1162–1166. 10.1038/nature04302 16372011 PMC1416283

[B41] Kaushalya S. K. Desai R. Arumugam S. Ghosh H. Balaji J. Maiti S. (2008). Three-photon microscopy shows that somatic release can be a quantitatively significant component of serotonergic neurotransmission in the mammalian brain. J. Neurosci. Res. 86, 3469–3480. 10.1002/jnr.21794 18709651

[B42] Kravitz E. A. (1988). Hormonal control of behavior: amines and the biasing of behavioral output in lobsters. Science 241, 1775–1781. 10.1126/science.2902685 2902685

[B43] Kuffler D. P. Nicholls J. Drapeau P. (1987). Transmitter localization and vesicle turnover at a serotoninergic synapse between identified leech neurons in culture. J. Comp. Neurol. 256, 516–526. 10.1002/cne.902560404 2435767

[B45] Leon-Pinzon C. Cercós M. G. Noguez P. Trueta C. De-Miguel F. F. (2014). Exocytosis of serotonin from the neuronal soma is sustained by a serotonin and calcium-dependent feedback loop. Front. Cell. Neurosci. 8:169. 10.3389/fncel.2014.00169 25018697 PMC4072984

[B46] Li X. Yu B. Sun Q. Zhang Y. Ren M. Zhang X.. (2018). Generation of a whole-brain atlas for the cholinergic system and mesoscopic projectome analysis of basal forebrain cholinergic neurons. Proc. Natl. Acad. Sci. U S A 115, 415–420. 10.1073/pnas.1703601115 29259118 PMC5777024

[B47] Livingstone M. S. Harris-Warrick R. M. Kravitz E. A. (1980). Serotonin and octopamine produce opposite postures in lobsters. Science 208, 76–79. 10.1126/science.208.4439.76 17731572

[B48] Ludwig M. Sabatier N. Bull P. M. Landgraf R. Dayanithi G. Leng G. (2002). Intracellular calcium stores regulate activity-dependent neuropeptide release from dendrites. Nature 418, 85–89. 10.1038/nature00822 12097911

[B49] Ludwig M. Stern J. (2015). Multiple signalling modalities mediated by dendritic exocytosis of oxytocin and vasopressin. Philos Trans. R Soc. Lond B Biol. Sci. 370:20140182. 10.1098/rstb.2014.0182 26009761 PMC4455751

[B50] Maity B. K. Maiti S. (2018). Label-free imaging of neurotransmitters in live brain tissue by multi-photon ultraviolet microscopy. Neuronal Signal 2:NS20180132. 10.1042/NS20180132 32714595 PMC7373235

[B51] Marcaggi P. Attwell D. (2004). Role of glial amino acid transporters in synaptic transmission and brain energetics. Glia 47, 217–225. 10.1002/glia.20027 15252810

[B52] Mendez J. A. Bourque M. J. Fasano C. Kortleven C. Trudeau L. E. (2011). Somatodendritic dopamine release requires synaptotagmin 4 and 7 and the participation of voltage-gated calcium channels. J. Biol. Chem. 286, 23928–23937. 10.1074/jbc.M111.218032 21576241 PMC3129174

[B53] Mendolesi J. (2018). Exosomes and ectosomes in intercellular communication. Curr. Biol. 28, R435–R444. 10.1016/j.cub.2018.01.059 29689228

[B54] Metea M. R. Newman E. A. (2006). Calcium signaling in specialized glial cells. Glia 54, 650–655. 10.1002/glia.20352 17006893 PMC2289783

[B55] Mouton P. R. Pakkenberg B. Gundersen H. J. Price D. L. (1994). Absolute number and size of pigmented locus coeruleus neurons in young and aged individuals. J. Chem. Neuroanat. 7, 185–190. 10.1016/0891-0618(94)90028-0 7848573

[B56] Munsch T. Deitmer J. W. (1992). Calcium transients in identified leech glial cells in situ evoked by high potassium concentrations and 5-hydroxytryptamine. J. Exp. Biol. 167, 251–265. 1634865 10.1242/jeb.167.1.251

[B57] Nair-Roberts R. G. Chatelain-Badie S. D. Benson E. White-Cooper H. Bolam J. P. Ungless M. A.. (2008). Stereological estimates of dopaminergic, GABAergic and glutamatergic neurons in the ventral tegmental area, substantia nigra and retrorubral field in the rat. Neuroscience 152, 1024–1031. 10.1016/j.neuroscience.2008.01.046 18355970 PMC2575227

[B58] Newman E. A. (2015). Glial cell regulation of neuronal activity and blood flow in the retina by release of gliotransmitters. Philos Trans R Soc. Lond B Biol. Sci. 370:20140195. 10.1098/rstb.2014.0195 26009774 PMC4455764

[B59] Nicholls J. G. Kuffler D. P. (1990). Quantal release of serotonin from presynaptic nerve terminals. Neurochem. Int. 17, 157–163. 10.1016/0197-0186(90)90138-j 20504616

[B60] Noguez P. Rubí J. M. De-Miguel F. F. (2019). Thermodynamic efficiency of somatic exocytosis of serotonin. Front. Physiol. 10:473. 10.3389/fphys.2019.00473 31214038 PMC6554442

[B61] Nusbaum M. P. Blitz D. M. Marder E. (2017). Functional consequences of neuropeptide and small-molecule co-transmission. Nat. Rev. Neurosci. 18, 389–403. 10.1038/nrn.2017.56 28592905 PMC5547741

[B62] Pál B. (2018). Involvement of extrasynaptic glutamate in physiological and pathophysiological changes of neuronal excitability. Cell Mol. Life Sci. 75, 2917–2949. 10.1007/s00018-018-2837-5 29766217 PMC11105518

[B63] Patel J. C. Witkovsky P. Avshalumov M. V. Rice M. E. (2009). Mobilization of calcium from intracellular stores facilitates somatodendritic dopamine release. J. Neurosci. 29, 6568–6579. 10.1523/JNEUROSCI.0181-09.2009 19458227 PMC2892889

[B64] Perea G. Navarrete M. Araque A. (2009). Tripartite synapses: astrocytes process and control synaptic information. Trends Neurosci. 32, 421–431. 10.1016/j.tins.2009.05.001 19615761

[B65] Piccolino M. Neyton J. Gerschenfeld H. M. (1984). Decrease of gap junction permeability induced by dopamine and cyclic adenosine 3’5’-monophosphate in horizontal cells of turtle retina. J. Neurosci. 4, 2477–2488. 10.1523/JNEUROSCI.04-10-02477.1984 6092564 PMC6564702

[B66] Puopolo M. Hochstetler S. E. Gustincich S. Wightman R. M. Raviola E. (2001). Extrasynaptic release of dopamine in a retinal neuron: activity dependence and transmitter modulation. Neuron 30, 211–225. 10.1016/s0896-6273(01)00274-4 11343656

[B67] Quentin E. Belmer A. Maroteaux L. (2018). Somato-Dendritic regulation of raphe serotonin neurons; a key to antidepressant action. Front. Neurosci. 12:982. 10.3389/fnins.2018.00982 30618598 PMC6307465

[B68] Rusakov D. A. Kullmann D. M. (1998). Extrasynaptic glutamate diffusion in the hippocampus: ultrastructural constraints, uptake and receptor activation. J. Neurosci. 18, 3158–3170. 10.1523/JNEUROSCI.18-09-03158.1998 9547224 PMC6792642

[B69] Südhof T. C. (2013). Neurotransmitter release: the last millisecond in the life of a synaptic vesicle. Neuron 80, 675–690. 10.1016/j.neuron.2013.10.022 24183019 PMC3866025

[B70] Sarkar B. Das A. K. Arumugam S. Kaushalya S. K. Bandyopadhyay A. Balaji J.. (2012). The dynamics of somatic exocytosis in monoaminergic neurons. Front. Physiol. 3:414. 10.3389/fphys.2012.00414 23133421 PMC3490137

[B71] Stewart R. R. Adams W. B. Nicholls J. G. (1989). Presynaptic calcium currents and facilitation of serotonin release at synapses between cultured leech neurones. J. Exp. Biol. 144, 1–12. 2549164 10.1242/jeb.144.1.1

[B72] Sun Y. Poo M. -M. (1987). Evoked release of acetylcholine from the growing embryonic neurons. Proc. Natl. Acad. Sci. U S A 84, 2540–2544. 10.1073/pnas.84.8.2540 3470810 PMC304690

[B73] Thorn P. Zorec R. Rettig J. Keating D. J. (2016). Exocytosis in non-neuronal cells. J. Neurochem. 137, 849–859. 10.1111/jnc.13602 26938142

[B102] Tobin V. Leng G. Ludwig M. (2012). The involvement of actin, calcium channels and exocytosis proteins in somato-dendritic oxytocin and vasopressin release. Front. Physiol. 3:261. 10.3389/fphys.2012.00261 22934017 PMC3429037

[B74] Tobin V. A. Ludwig M. (2007). The role of the actin cytoskeleton in oxytocin and vasopressin release from rat supraoptic nucleus neurons. J. Physiol. 582, 1337–1348. 10.1113/jphysiol.2007.132639 17478532 PMC2075266

[B75] Trueta C. De-Miguel F. F. (2012). Extrasynaptic exocytosis and its mechanisms: a source of molecules mediating volume transmission in the nervous system. Front. Physiol. 3:319. 10.3389/fphys.2012.00319 22969726 PMC3432928

[B76] Trueta C. Kuffler D. P. De-Miguel F. F. (2012). Cycling of dense core vesicles involved in somatic exocytosis of serotonin by leech neurons. Front. Physiol. 3:175. 10.3389/fphys.2012.00175 22685436 PMC3368391

[B77] Trueta C. Méndez B. De-Miguel F. F. (2003). Somatic exocytosis of serotonin mediated by L-type calcium channels in cultured leech neurones. J. Physiol. 547, 405–416. 10.1113/jphysiol.2002.030684 12562971 PMC2342656

[B103] Trueta C. Sánchez-Armass S. Morales M. A. De-Miguel F. F. (2004). Calcium-induced calcium release contributes to somatic secretion of serotonin in leech Retzius neurons. J. Neurobiol. 61, 309–316. 10.1002/neu.20055 15389693

[B78] Umbriaco D. Garcia S. Beaulieu C. Descarries L. (1995). Relational features of acetylcholine, noradrenaline, serotonin and GABA axon terminals in the stratum radiatum of adult rat hippocampus (CA1). Hippocampus 5, 605–620. 10.1002/hipo.450050611 8646286

[B79] Verkhratsky A. Krishtal O. A. Burnstock G. (2009). Purinoceptors on neuroglia. Mol. Neurobiol. 39, 190–208. 10.1007/s12035-009-8063-2 19283516

[B80] Willard A. L. (1981). Effects of serotonin on the generation of the motor program for swimming by the medicinal leech. J. Neurosci. 1, 936–944. 10.1523/JNEUROSCI.01-09-00936.1981 7288474 PMC6564106

[B81] Witkovsky P. Patel J. C. Lee C. R. Rice M. E. (2009). Immunocytochemical identification of proteins involved in dopamine release from the somatodendritic compartment of nigral dopaminergic neurons. Neuroscience 164, 488–496. 10.1016/j.neuroscience.2009.08.017 19682556 PMC2879289

[B82] Witkovsky P. (2004). Dopamine and retinal function. Doc. Ophthalmol. 108, 17–40. 10.1023/b:doop.0000019487.88486.0a 15104164

[B83] Zetler G. (1970. “Distribution of peptidergic neurons in mammalian brain,” in Aspects of Neuroendocrinology, eds Bargmann W. Scharrer B., 287–295. 10.1007/978-3-642-46207-8_30

[B84] Zhang A. J. Jacoby R. Wu S. M. (2011). Light- and dopamine-regulated receptive field plasticity in primate horizontal cells. J. Comp. Neurol. 519, 2125–2134. 10.1002/cne.22604 21452210 PMC3632401

